# Factors Affecting the Differentiation of The Apgar Score and The Biochemical Correlation of Fetal Well-being – a Prospective Observational Clinical Study

**DOI:** 10.34763/devperiodmed.20182203.238246

**Published:** 2018-10-04

**Authors:** Maria Kostro, Natalia Jacyna, Ewa Głuszczak-Idziakowska, Katarzyna Sułek-Kamas, Grzegorz Jakiel, Maria Wilińska

**Affiliations:** 1 Klinika Neonatologii SPSK im. prof. W. Orłowskiego CMKP, Warszawa, Polska; 2 I Klinika Ginekologii SPSK im. prof. W. Orłowskiego CMKP, Warszawa, Polska

**Keywords:** term newborn, neonatologist, Apgar score, umbilical cord blood, noworodek donoszony, neonatolog, skala Apgar, krew pępowinowa

## Abstract

**Objective:**

The purpose of the study was to identify the features of both the labor and the assisting physicians when evaluating the newborn according to the Apgar score and how these correlate with the biochemical markers of fetal well-being in order to make the Apgar score more objective.

**Material and methods:**

A prospective observational clinical study conducted in a 3^rd^ reference level center between 1^st^ April 2014 and 31^st^ March 2015. The study enrolled 17 neonatologists and 1527 term newborns.

**Results:**

The Apgar score is highest after natural vaginal delivery, lower after instrumental labor (p <0.001). The pH of the umbilical cord blood and lactate concentration correlate better with a high score than with a lowered one. The young age of a physician does not reduce Apgar score reliability. There were no differences in Apgar assessment according to physicians’ training and the time of labor. There were no correlations between abnormalities in postnatal central nervous system ultrasound and the Apgar score.

**Conclusion:**

Biochemical tests of umbilical cord blood significantly increase the Apgar score reliability.

## Streszczenie

Skala Apgar jest powszechnie używana dla oceny noworodka po urodzeniu, jednak wydaje się konieczne ustalenie czynników zmniejszających subiektywizm tej punktacji.

**Cel:**

Identyfikacja cech porodu oraz lekarzy oceniających noworodka po urodzeniu według skali Apgar oraz jej korelacja z wykładnikami biochemicznymi dobrostanu płodu.

**Materiał i metody:**

Prospektywne obserwacyjne badanie kliniczne przeprowadzone w ośrodku III poziomu referencyjnego, w okresie 01.04.2014-31.03.2015. Badaniem objęto grupę 17 lekarzy neonatologów oraz 1527 donoszonych noworodków.

**Wyniki:**

Punktacja Apgar jest najwyższa po porodzie naturalnym, niższa po porodach zabiegowych (p<0,001). Z pH krwi pępowinowej i stężeniem mleczanów lepiej koreluje punktacja wysoka niż obniżona. Młody wiek lekarza nie zmniejsza wiarygodności oceny wg Apgar. Nie wykazano różnic w ocenie Apgar uwarunkowanych stopniem wyszkolenia lekarzy oraz porą porodu. Nie wykazano korelacji nieprawidłowości w obrazie USG głowy z punktacją po urodzeniu. W opracowaniu wyników użyto test niezależności chi^2^.

**Wnioski:**

Wiarygodność skali Apgar u noworodków donoszonych istotnie podwyższają badania biochemiczne krwi pępowinowej.

## Introduction

An objective assessment of the newborn right after birth is desirable for the implementation of appropriate medical procedure to stabilize the clinical condition as well as predict the future medical needs of a child. It is also a summary and success assessment of obstetrician personnel, especially in the eyes of laboring women.

More than 60 years ago, Virginia Apgar proposed a score that allowed rapid and easy assessment of newborns’ condition after birth [1]. The scale is based on five parameters: heart rate, respiratory effort, muscle tone, reflex irritability and skin colour. These parameters are evaluated in the 1st, 3rd, 5th and 10th minute of life. Each parameter is assessed as “0” in case of significant irregularities, 2 points in good condition and 1 point in the case of indirect assessment. The sum of points equaling 8-10 means good condition, 4-7 points − average condition and 0-3 points - bad condition.

Criticism arose together with the introduction of the score. There were those who proposed their own child assessment systems or biochemical alternatives to clinical evaluation. The main objection to Apgar is its subjectivity. Only two of the five criteria are measurable − the number of breaths and the heart rate. Despite many attempts so far, it has not been possible to clearly identify factors that cause a different assessment of the same newborn by those involved in childbirth [2, 3 ,4, 5].

As early as in the first century AD there was a score elaborated by Soranus of Ephesus, which assessed the survival chances of newborns. This evaluation system was very similar to the Apgar score. Soranus of Ephesus included very important points in his system, which, according to critics, Virginia Apgar missed. These were: the presence of congenital disorders in the fetus, the maturity of the fetus and the condition of the pregnant woman’s health. There is no evidence that Virginia Apgar knew the score of Soranus from Ephesus and it is unlikely [6].

Apgar score critics have been trying to modify this neonatal assessment system. The A-C score was proposed (“Apgar minus Color”), which omits the neonatal skin color criterion, as the least important and most subjective [7]. However, this idea has not gained widespread acceptance.

Other modern scores for evaluating newborns include SNAP (The Score for Neonatal Acute Physiology) and CRIB (Critical Risk Index for Babies). Both of them are used for estimating the risk of newborn death based on the baby’s physical examination and blood gasometry. Unfortunately they have a fairly complicated layout and omit healthy newborns and those whose condition quickly improved [8, 9].

In 2006, the AAP (American Academy of Pediatrics) introduced the expanded Apgar score, which includes resuscitation parameters performed in the delivery room. This modification identifies the oxygen supply data, the type of respiratory support (NPPV, NCPAP), endotracheal intubation and mechanical ventilation, external cardiac massage and adrenaline use [10].

Despite many years of using the Apgar score, it is still not quite clear why various people evaluate the same newborn differently right after birth. Identification of these factors could help to make the score objective. It also seems appropriate to periodically verify the credibility of the Apgar score used by new generations of neonatologists.

### Purpose

The purpose of the study was to identify the characteristics of neonatologists and factors related to childbirth which influence the differentiation of neonatal assessment after birth according to the Apgar score. In addition, the correlation between this assessment and the biochemical determinants of fetal wellbeing was examined.

## Material and methods

Term newborns (≥37 weeks gestation) without major birth malformations significantly affecting the clinical condition (lung, heart, digestive tract, skeletal system, diaphragm malformations), were eligible for the study with parental consent. Newborns who did not meet the above criteria were not included in the study.

The data was collected retrospectively by the neonatologist who participated in the childbirth. The test form included the demographic and clinical data of the child (gestational age, body weight, Apgar score in the 1st minute), information about childbirth (natural birth, caesarean section, time of delivery) and characteristics of the physician (age, work experience, specialization).

After clamping the umbilical cord on two sides, the person responsible for laboratory testing had to identify the vessels and take blood from the umbilical artery to the heparinized capillary (up to 10 minutes after delivery). Gasometry should take place immediately after taking a blood sample (up to 10 minutes) [11]. Selected parameters of acid-base balance were evaluated, such as pH, BE basic excess) and lactate concentration. Biochemical criteria of fetal asphyxia was pH<7.20, BE ≥ (-10) and lactic acid concentration >60 mg/dl [11].

Childbirth finished between 7:00 and 18:59 was classified as taking place during the day, while it was considered nocturnal if it was completed between 19:00 - 06:59.

On the second day of life, a central nervous system (CNS) cranial ultrasound was performed in each child. This study was performed for potential intraventricular haemorrhage (IVH), periventricular leukomalacia (PVL), congenital malformations, hypoxic ischemic lesions, and sinus sagittal thrombosis.

In our study, we took into consideration only the evaluation in the 1st minute of life of the newborn, as only this score correlates with the biochemical tests of umbilical cord blood [3, 12]. The Apgar scores in the following minutes are rather an indicator of the effectiveness of neonatal resuscitation [3, 13].

Statistical analysis was performed using the Stata®/ Special Edition ver. 14 program. Univariate analysis and multivariate analysis was performed using the Chi-squared test of independence, ANOVA without replication, binary logistic regression, ordinal regression, mixed-effects logistic regression and multinomial regression.

The approval for conducting this study was granted by the Bioethics Committee at the Postgraduate Medical Education Center.

## Results

The study was conducted at the Public Clinical Hospital Center of Medical Postgraduate Education in Warsaw (3rd level of perinatal care), from 1^st^ April 2014 to 31^st^ March 2015.

**Fig. 1:**
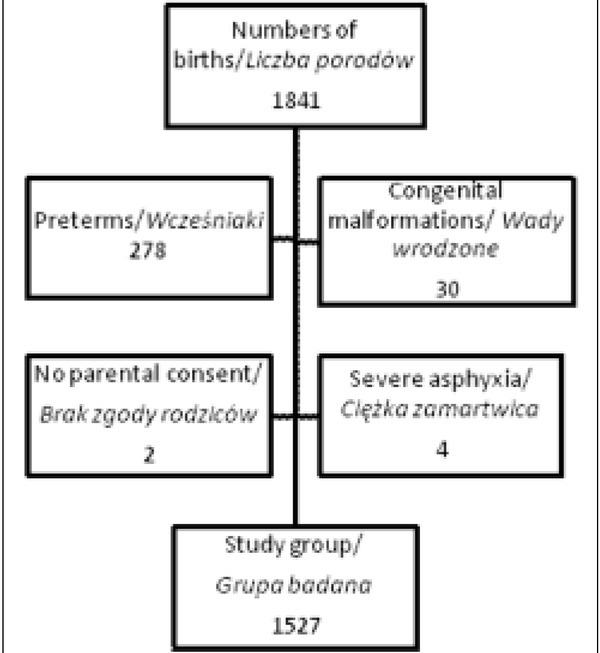
The qualification scheme for the study. Ryc. 1. Schemat kwalifikacyjny badania.

1841 deliveries took place in the hospital during the study. Preterm infants (n=287), with congenital malformations (n=30), in severe asphyxia (n=4), and those whose parents did not give their consent (n=2) were excluded from the study. 1527 patients were enrolled for further study (Figure 1).

The study involved 17 physicians, mainly women (88%), aged 27-63 (median 30 years). 99% of the questionnaires (n=1512) were filled by women and 1% (n=15) by men. 34% of the physicians conducting assessment had completed their specialization in neonatology, the others were physicians during specialization.

The survey population included an equal percentage of male and female newborns. The median maturity was 39 weeks of pregnancy, body weight 3426 g. The majority of newborns were born by normal vaginal delivery (nvd), 40% by caesarean section (cs), 2% by vacuum extractum (ve). Only 3% of the newborns (n=40) were born in an average overall condition, with a score of 4 to 7 points in the 1st minute of life, the rest received high scores: 8 and more points. A slightly higher number of children were born during the day (Table I).

**Table I:** The demographic characteristics of the examined groups of physicians and newborns. Tabela I. Charakterystyka demograficzna lekarzy wypełniających ankietę oraz badanych noworodków.

	Feature/*Cecha*	Value/*Wartość*
**Physicians/*Lekarze* (n=17)**		
	Male/*Mężczyźni* (%)	2 (12)
	Number of questionnaires*/ /Liczba wypełnionych ankiet*	15 (1)
	
	Female/*Kobiety* (%)	15 (88)
	*Number of questionnaires/ /Liczba wypełnionych ankiet*	1512(99)
	
	Age (years)/*Wiek* (lata)	30 * (27 - 63)
	
	Specialization (%)/ *Posiadana specjalizacja*	9 (53)
	Number of questionnaires *Liczba wypełnionych ankiet*	511 (34)

**Newborns/*Noworodki* (n=1527)**		
	Male/*Płeć męska* (%)	763 (50)
	
	Female/*Płeć żeńska* (%)	764 (50)
	
	Birthweight (grams)/Masa ciała (gramy)	3426* (2050-5100)
	
	Gestation at birth (weeks)/ */Dojrzałość dziecka (hbd)*	39 (37-41)
	
	Delivery/*Rodzaj porodu*	
	
	nvd*/psn (%)*	886 (58)
	
	cs*/cc (%)*	611 (40)
	
	ve *(%)*	30 (2)
	
	Apgar in 1 minute/ *Punktacja Apgar w 1’*	
	8-10 points/*8-10 punktów (%)*	1480 (97)
	4-7 points/*4-7 punktów (%)*	49(3)
	
	Time of birth/*Pora dnia porodu*	
	During the day*/W ciągu dnia −* 07:00-18:59 *(%)*	863 (56,5)
	At night*/W nocy −* 19:00-06:59 *(%)*	664(43,5)

Legend: cs – caesarian section, nvd – natural vaginal delivery, ve – vacuum extractor *median Legenda: cc – cięcie cesarskie, psn – poród siłami natury, ve – vacuum extractor. *mediana

Studying the correlation between the degree of the physician’s training and neonatal evaluation showed that neonatal specialization did not affect the Apgar score after birth (p=0.255). Moreover, in the survey there was no correlation with the performing neonatologist’s age (p=0.93), which means that younger and older physicians of the neonatal team made similar evaluations. There was no correlation between the time of labor and the Apgar score (p=0,297).

**Fig. 2:**
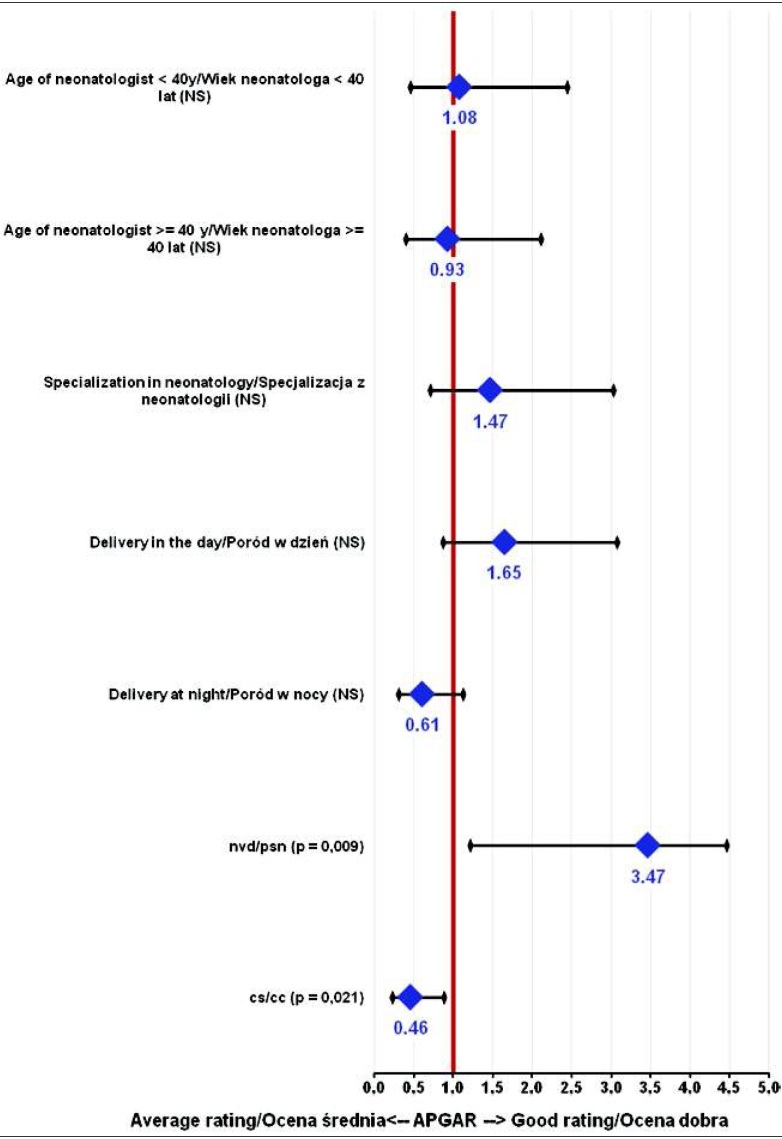
Correlation between Apgar score and the features of both the evaluators and labor. Ryc. 2. Analiza zależności między cechami osób oceniających i charakterystyką porodu a punktacją Apgar. Legend: y – years, NS – not significant, nvd – natural vaginal delivery, cs – caesarian section Legenda: NS – nieistotne statystycznie, psn – poród siłami natury, cc – cięcie cesarskie

Infants born by nvd (p=0.009) were given a higher score in Apgar than those delivered by cs (p=0.021) and with the use of vacuum (p<0.001) (Figure 2).

Cord arterial blood gasometry was tested in 1272 cases out of 1527 infants born and qualified for the survey. The good condition according to Apgar was confirmed in 92.21% by good pH results and in 98.86% by normal BE result. A weaker correlation was found in the group of neonates evaluated as average. In this group 52.5% had abnormal pH and only 20% had BE deficiency.

1214 children were tested for lactic acid concentration. Good postnatal status was confirmed in 83.6% of the group with normal levels of lactic acid. 51.35% of the patients with an average score had elevated lactate levels.

1203 newborns were subjected to cranial ultrasound. Changes in CNS were not reported in 88.15% of patients born in good condition. Irregularities of the ultrasound examination occurred in 140 patients (11% of the group). Only two of them were born in an average general condition (5.26%). The most common condition was IVH I ° (90 cases) followed by lateral ventricular dilatation (18 cases) and increased echogenicity of the periventricular area (15 cases). Neonates did not experience grade III or IV haemorrhage or sagittal sinus thrombosis in the study group.

There was no correlation between decreased Apgar score and abnormalities in the CNS (p=0.232) (Table II).

**Table II:** The biochemical results and the type of abnormality detected by CNS examination. Tabela II. Wyniki biochemiczne oraz rodzaj nieprawidłowości w usg OUN.

Biochemical results *Wynik biochemiczny*		Newborn condition according to the Apgar score *Stan noworodka wg skali Apgar*					
		Good condition *Stan dobry*		Average condition *Stan średni*		Χ2 test result *Wynik testu χ2*	p-value *p*
		N % of the group	N	% of the group		
pH	Normal/*Prawidłowe*	1136 92.21	19	47.5	87.44	<0.001
	Abnormal/*Nieprawidłowe*	96 7.79	21	52.5		
BE	Normal/*Prawidłowe*	1218 98.86	32	80	70.26	<0.001
	Abnormal/*Nieprawidłowe*	14 1.14	8	20		
Lactate/ /*Mleczany*	Normal/*Prawidłowe*	984 83.6	18	48.65	30.41	<0.001
	Abnormal/*Nieprawidłowe*	193 16.4	19	51.35		
USG	Normal/*Prawidłowe*	1027 88.15	36	94.74	0.98	0.323
	Abnormal/*Nieprawidłowe*	138 11.85	2	5.26		
	IVH I**°**	88 62	2	1		
	IVH II **°**	10 7	-	-		
	PVL	3 2	-	-		
	Ventricular dilatation *Poszerzenie układu komorowego*	18 13	-	-		
	CNS malformation not *Wada* diagnosed *OUN nie* in *wykryta* utero *prenatalnie*	4 3	-	-		
	Increased echogenicity of perivenrticular area *Wzmożona echogeniczność okolicy okołokomorowej*	15 11	-	-		

Legend: BE – Base excess, CNS – central nervous system, IVH – intraventricular haemorrhage, PVL – periventricular leukomalacia Legenda: BE – niedobór zasad, IVH – krwawienie dokomorowe, OUN – ośrodkowy układ nerwowy, PVL – leukomalacja okołokomorowa

Analyzing the correlation between Apgar scores and biochemical findings with the physician’s age revealed that the assessments performed by young physicians were more in line with the biochemical tests (p=0.025) compared to older neonatologists. In addition, there was a higher compliance of the clinical evaluation with biochemical tests for natural births (p=0.025) compared to delivery by caesarean section.

**Fig. 3:**
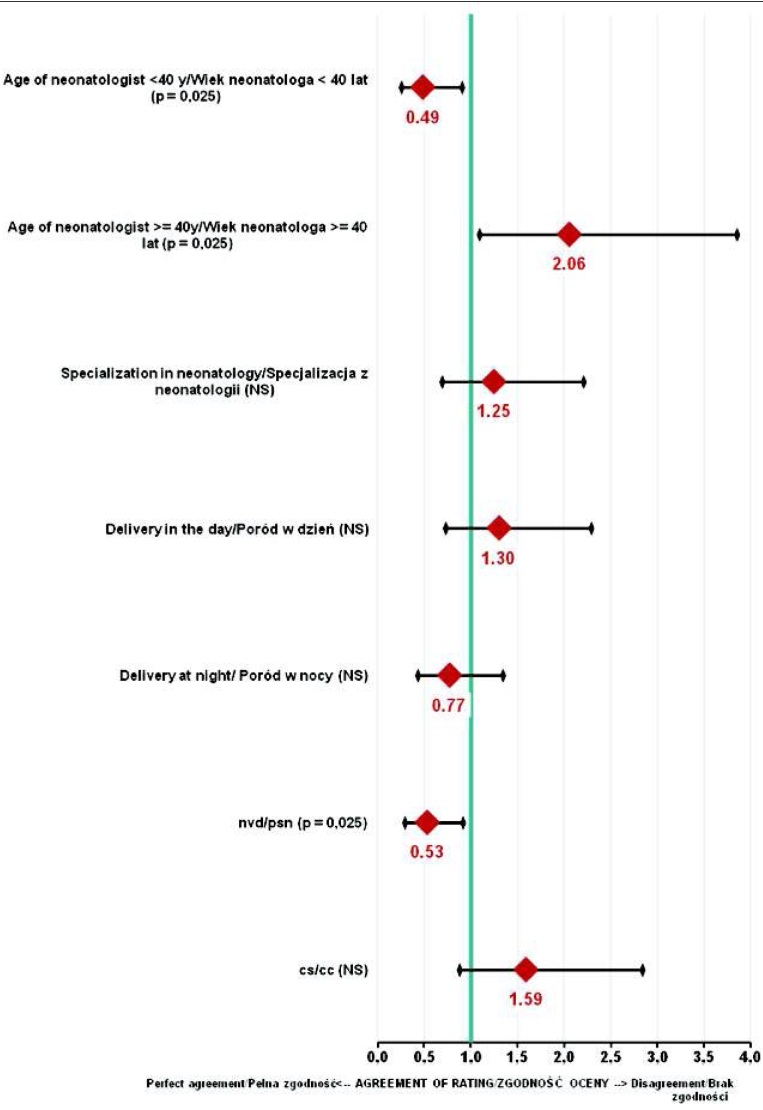
Correlation of Apgar score and biochemical findings with the features of the evaluators and labor Characteristics. Ryc. 3. Analiza zależności między oceną Apgar potwierdzoną badaniami biochemicznymi a cechami osób oceniających i rodzajem porodu. Legend: y – years, NS – not significant, nvd – natural vaginal delivery, cs – caesarian section Legenda: NS – nieistotne statystycznie, psn – poród siłami natury, cc – cięcie cesarskie

The specialization of the physician, assisted vaginal delivery or caesarian section and the time of birth did not influence the increase or decrease of assessment conformity with biochemical parameters (Figure 3).

## Discussion

The Apgar score, despite its widespread use, still raises a lot of emotions among neonatologists. It is also a subject of their disputes with obstetricians.

However, there are many arguments that confirm the fact that the Apgar score is still the best tool for assessing neonatal status right after birth and for estimating the risk of neonatal death [3, 10, 14. It has been documented that a low Apgar score is a better predictor of child death than pH<7.0 [14].

Unfortunately, the Apgar score does not allow us to estimate the risk of late mortality [15] or the labor influence on future psychomotor development or cerebral palsy [10, 15, 16].

It must be taken into account that Virginia Apgar’s score does not include medication given to pregnant women, neonatal maturity or resuscitation [10].

According to the latest international study that involves countries reporting to the Euro – Peristat [17] there are large differences in the Apgar score of healthy newborns between different countries and regions. This variation is most probably due to the presence of local habits of assessing healthy newborns that are passed on to younger generations of physicians. Therefore, it is not possible to compare the condition of the population among different nations on the basis of Apgar scores, but it is possible to observe the improvement or deterioration of health within a nation.

At the same time, the authors conclude the need to identify external factors influencing the differentiation of Apgar’s assessment by physicians.

Not all Polish neonatologists trust the Apgar score. 88.5% of them think that it is of little value [4]. In spite of that, current Polish recommendations do not foresee the supplementation of Apgar assessment of the baby after birth with a biochemical examination of umbilical cord blood [18]. On the other hand, the Polish Gynecological Society recommends testing acid-base balance and lactic acid as the only objective parameters of fetal well-being [19].

In practice fetal welfare monitoring during childbirth varies across the world. In the United Kingdom, Germany, Australia or Sweden if the CTG reading is inappropriate, it is recommended to take blood from the fetal scalp in order to determine the level of lactic acid as the best marker for obstetric failure [20, 21]. French recommendations provide for both the artery and the umbilical vein to be sampled [22]. “

According to the AAP, Apgar score should not be the only evidence of neurological damage resulting from labor events. The diagnosis of perinatal hypoxia should be based on documented anomalous gasometry which should be performed whenever the Apgar score in the 5th minute is less than or equal to 5 [23].

In order to make our study objective, we took into account umbilical cord blood gasometry and the concentration of lactic acid [24]. A correlation has been established between lactic acidosis in neonates and hypoxia [25]. Moreover, lactic acid concentration is considered to be the first indicator of hypoxia in the body, before decreased pH and elevated BE [26].

Based on the above study, it has been shown that the Apgar score correlates with the type of childbirth. The highest scores were given to newborns born by natural vaginal delivery, slightly lower ones to those born by caesarean section, and the lowest to those subjected to assisted delivery with a vacuum extractor. This is in line with the study of V. Apgar [27]. According to her, the clinical condition of the infants after breech presentation is the worst, slightly better is the condition of those born by caesarean section, and infants delivered by nvd are in the best condition.

We have not found that the number of Apgar points awarded was dependent on any of the physician’s attributes (neonatal specialization, age of the physician evaluating the child), or on the time of birth − whether the child was born during the daytime or during nighttime in artificial lighting.

O’Donnell also failed to show why different physicians rate the same child differently. Differences in evaluation did not depend on race, lighting (day/night), age of pregnancy, or the “thickness” of neonatal subcutaneous tissue [2]. He believes, however, that “ various physicians’ characteristics influence the assessment of the newborns’ skin color rather than the differences between the newborns themselves.” In the conclusion of his research O’Donnell states that in order to objectively assess the baby’s oxygenation after birth, only a pulse oximeter should be used.

In our study we have shown a very high correlation between a good Apgar score and normal pH, BE and lactate levels (>90% of patients). The average Apgar score was confirmed by abnormal pH and lactate results in over 50% of newborns. Many other studies confirm this phenomenon [13, 28].

We also found that the Apgar score awarded by young physicians (<40 years) was more in line with laboratory tests. Such compliance also occurred when natural vaginal delivery took place. No similar claims have been found so far in the literature.

The average Apgar score did not correlate with abnormalities in cranial ultrasound of CNS in the examined neonatal group. It means that the Apgar score does not determine the necessity of cranial ultrasound in newborns.

## Conclusions

In a medical team with standardized procedures, neonatal evaluation according to Apgar score does not depend on the human factor. Childbirth with a vacuum extractor and caesarean section is associated with poorer postnatal assessment. This may be due to earlier existing fetal well-being disorders, drugs administered to pregnant women, or the lack of positively stimulating perinatal stress in elective caesarian sections.

We have shown a higher compliance of the Apgar score with biochemical tests for younger physicians in comparison to the group of older doctors, but this observation requires further investigation. The assessment of a newborn according to the Apgar score usually correlates well with the results of biochemical markers of umbilical arterial blood. It should be added that higher compliance is observed for neonates with a good Apgar score, while lower compliance is observed for those born in an average general condition.

Based on the above observations, we conclude that for both complete and objective evaluation of fetal and term neonate well-being, it is most desirable to combine both methods (Apgar score and biochemical evaluation), which should remain standard practice at a labor ward.
